# Reproductive seasonality influences follicle dynamics and the ovarian extracellular matrix structural properties in ewes

**DOI:** 10.1530/REP-25-0010

**Published:** 2025-05-22

**Authors:** Johanne Grosbois, Vlastimil Srsen, Alba Muñoz Grande, Helen M Picton, Evelyn E Telfer

**Affiliations:** ^1^ Institute of Cell Biology, Hugh Robson Building, The University of Edinburgh, Edinburgh, UK; ^2^ Centre for Reproductive Health, Institute of Regeneration and Repair, The University of Edinburgh, Edinburgh, UK; ^3^ Reproduction and Early Development Research Group, Discovery and Translational Science Department, Leeds Institute of Cardiovascular and Metabolic Medicine, School of Medicine, University of Leeds, Leeds, UK

**Keywords:** ovary, seasonal reproduction, extracellular matrix, photoperiod, mechanobiology

## Abstract

**In brief:**

Although sheep have been widely used as a large animal model for human ovarian biology, unlike women, they display a marked seasonality of breeding activity, the underlying mechanisms and extent of ovarian changes of which remain largely undefined. This study reveals the active remodeling of the ovarian extracellular matrix across the reproductive season, which could be an additional driver responsible for the observed variations in ovarian morphometry and follicle dynamics.

**Abstract:**

Ovarian function requires dynamic tissue remodeling provided by its extracellular matrix (ECM). In seasonal breeders, ovaries undergo an additional circannual cycle of recrudescence and regression. While increasing evidence suggests that the ECM impacts normal ovarian cyclicity and function, how its components are remodeled across reproductive seasonality has not been explored in large mammals. Using immunohistological and *in vitro* experiments, we investigated the influence of reproductive seasonality on ovarian morphometry, ECM properties and follicle developmental potential *in vitro*. Ovarian weight and volume were reduced during anestrus (*P* < 0.001). Neither follicular density nor the proportion of preantral follicles and earlier stages of development were impacted by the season, but the percentage of antral follicles increased during anestrus (*P* = 0.028), while corpora lutea were only present in ovaries collected during the breeding season. Concomitantly, ovarian ECM composition was significantly remodeled, with stromal collagen and fibronectin significantly increased (*P* < 0.01) and laminin decreased (*P* = 0.032) during anestrus compared to the breeding season. This correlated with thicker collagen fibers both in the stroma and in the tunica albuginea during anestrus. *In vitro*, preantral follicles isolated from their native environment exhibited a season-dependent pattern of follicular integrity, survival, antrum formation and growth. These results suggest the establishment of a stiffer ovarian microenvironment during anestrus, which, together with endocrine changes, regulates follicle growth, demise and the ovulatory response.

## Introduction

The functional unit of the ovary is the ovarian follicle, composed of an oocyte surrounded by layers of supporting somatic granulosa cells. The process of follicle growth and development, known as folliculogenesis, is a complex and tightly coordinated process that depends on the bidirectional communication between the oocyte and the somatic-derived granulosa and theca cells, and ultimately leads to the generation of a developmentally competent oocyte (Li & Albertini 2013). In sheep, follicle development can be divided into gonadotropin-independent, gonadotropin-responsive and gonadotropin-dependent phases. While early folliculogenesis is primarily directed by paracrine factors produced within the local environment, preantral and small-to-medium antral follicles become increasingly responsive to pituitary gonadotropins (luteinizing hormone – LH and follicle-stimulating hormone – FSH), whose actions are modulated by local growth factors. The terminal stages of follicle development remain primarily under the control of the pituitary gonadotropins, with both FSH and LH having essential roles in regulating the final maturation and selection of the ovulatory follicle (Scaramuzzi *et al.* 2011). At the time of ovulation, the LH surge triggers a series of remodeling events within the preovulatory follicle wall that culminates in follicular rupture and the release of the oocyte (Telfer *et al.* 2023).

Successful ovarian function, which includes follicular development and degeneration, ovulation, and corpus luteum formation and regression, requires extensive cyclical tissue remodeling provided by its extracellular matrix (ECM) (Ny *et al.* 2002, Smith *et al.* 2002). The ovarian ECM is a cell surface-associated macromolecular network that forms the 3D scaffold in which follicles and stromal cells reside. Its components, both within and outside of the follicles, are dynamically expressed throughout follicle development and influence cell communication, proliferation, differentiation and survival (Huet *et al.* 2001, Woodruff & Shea 2007). The ovarian ECM was previously considered to be a passive structure that provides anchoring of matrix-bound growth factors and mechanical support to the cells, but it is now recognized as a pivotal element in the multidirectional communication that occurs between components of the developing follicle, i.e. ECM, stroma, theca, granulosa cells and the oocyte (Woodruff & Shea 2007). Notably, the composition of the ovarian ECM has been shown to undergo significant changes during follicular development (Ouni *et al.* 2020). The deposition and structural organization of ECM molecules determine the stiffness of the perifollicular environment, which affects the balance between maintenance of quiescence versus entry into growth of primordial follicles (Xu *et al.* 2006, Nagamatsu *et al.* 2019). ECM-related proteolytic enzymes also remodel the local environment during follicle expansion and ovulation (Curry *et al.* 2003), and folliculogenesis-regulating growth factors are sequestered within the ECM (Logan & Hill 1992).

Given the limited availability of human ovarian tissue for research purposes, there is an unmet need for a robust animal model beyond the mouse, comparable with women. Previous studies have highlighted the value of domesticated livestock animals, including sheep, in improving our understanding of ovarian biology (Gosden *et al.* 1994, Campbell *et al.* 2000, Campbell *et al.* 2003, Forsdike *et al.* 2007, Campbell *et al.* 2014). Ewes share similar biochemical, physiological and anatomical reproductive features with women, including folliculogenesis waves and ovarian compartmentalization, with primordial follicles distributed superficially in the cortex (Fransolet *et al.* 2014), but they are seasonal breeders, with their reproductive activity being regulated by the photoperiod. Briefly, light is sensed by the retina and then neurally transmitted to the pineal gland, which secretes melatonin. Melatonin secretion fluctuates with photoperiod changes to regulate reproductive endocrine function through the hypothalamic–pituitary axis by controlling the frequency of pulsatile secretion of gonadotropin-releasing hormone (GnRH) and LH, and subsequent secretion of gonadotropins (FSH and LH) (Nishiwaki-Ohkawa & Yoshimura 2016, Dardente & Simonneaux 2022). In most sheep breeds in temperate climates from the Northern Hemisphere, this is translated into a sexually active status from mid-autumn into winter and a sexually inactive status (anestrus) from spring through to autumn (Dardente *et al.* 2016). This also means that their ovaries undergo an additional circannual cycle of recrudescence and regression.

While components of the ECM impact normal ovarian cyclicity, how these elements are remodeled across reproductive seasonality has not been explored in sheep. The aim of the present study was to investigate the influence of reproductive seasonality on follicle dynamics and the structural properties of the ovarian ECM.

## Materials and methods

### Sample collection

Sheep ovaries were obtained from a local abattoir and transported in HEPES-buffered tissue culture medium 199 pre-warmed at 37°C and supplemented with amphotericin B (2.5 μg/mL), sodium pyruvate (25 μg/mL), penicillin G (75 μg/mL) and streptomycin (50 μg/mL; all chemicals obtained from Sigma-Aldrich, UK), as previously described (Sakaguchi *et al.* 2023). Ovaries were collected between November and January (breeding season) and between April and June (anestrus). While unable to control for the specific breed from which the ovaries were harvested, the company primarily processes Texel and Scottish Blackface sheep breeds as well as Texel/Scottish Blackface crossbreds. At the laboratory, ovaries were rinsed in saline solution, weighed and measured. Sixteen ovaries (eight per experimental group) obtained from four collections (*n* = 4 ovaries per collection) were sagittally halved using a scalpel and fixed in 10% normal buffered formalin for histological and immunohistological evaluation; the remaining ovaries, obtained from a total of 11 collections (two during the breeding season; four during early anestrus – mid-April to mid-May; and five during late anestrus – mid-May to the end of June), were used for preantral follicle isolation and culture.

### Histological analysis

Fixed hemi-ovaries were dehydrated in increasing concentrations of ethanol (70–100%, v/v), embedded in paraffin and serially sectioned at 7 μm thickness. Four consecutive tenth sections, distanced 70 μm apart and collected from the center of the ovary (largest surface), were stained with either hematoxylin and eosin or Picrosirius Red (PSR). Follicular density and developmental stage were assessed morphologically. The number of follicles per section was calculated by dividing the total number of follicles in four sections by 4. Follicle density was calculated by dividing the number of follicles in four sections by the volume of ovarian tissue analyzed, measured as the surface of those four sections (ImageJ) multiplied by section thickness. Follicles were classified according to their developmental stage as previously described (Yano Maher *et al.* 2024): primordial follicles (oocyte surrounded by a few flattened granulosa cells), transitional primordial follicle (oocyte surrounded by flattened and at least one cuboidal granulosa cell), primary follicles (oocyte surrounded by one complete layer of cuboidal granulosa cells), preantral follicles (oocyte surrounded by two or more complete layers of cuboidal granulosa cells), antral follicles (presence of an antrum) and the presence of a corpus luteum was recorded.

For PSR staining, four sections were deparaffinized and rehydrated in a series of ethanol baths of decreasing concentrations. The slides were immersed in a PSR staining solution (ab246832, Abcam, UK) for 1 h at room temperature, then washed twice with 0.5% (v/v) glacial acetic acid and three times with 100% ethanol. The slides were cleared in xylene and mounted with DPX. All slides from the same ovary were processed at the same time to minimize staining variation.

### Immunostaining

Four consecutive tenth sections, distanced 70 μm apart and collected from the center of the ovary (largest surface), were immunostained with fibronectin (FN-1) or laminin β2 (LAMB2). Briefly, after deparaffinization and rehydration, slides were immersed in Tris–EDTA buffer (pH 8.5) (Sigma-Aldrich) for 40 min at 95°C, then immersed in 3% (v/v) hydrogen peroxide (Sigma-Aldrich) to quench endogenous peroxidase activity. Non-specific binding sites were blocked using 5% normal goat serum for 1 h at room temperature. Sections were subsequently incubated overnight at 4°C for Anti-FN1 (Atlas Antibodies, Sigma-Aldrich, cat#HPA027066, 1:200) or Anti-LAMB2 (Atlas Antibodies, Sigma-Aldrich, cat#HPA001895, 1:100) antibodies. The next day, slides were incubated for 1 h at room temperature with a biotinylated secondary antibody (1:500), rinsed twice, and then incubated with Streptavidin Horseradish Peroxidase (Vectastain Elite ABC kit, Vector Laboratories, UK) for 30 min at room temperature. After washing, the signal was visualized using 3,3′-diaminobenzidine, then counterstained with hematoxylin, dehydrated and mounted with DPX. Negative controls were obtained by replacing the primary antibody with blocking solution containing non-immune serum. All washes were performed in PBS or PBS with 0.05% (v/v) Triton X-100 at room temperature. All slides from the same ovary were processed at the same time to minimize staining variation.

### Image analysis

Whole ovarian sections were imaged using an Axioscan slide scanner and birefringent images were acquired using an Axiovert 200 microscope (Carl Zeiss, Germany). The thickness of the tunica albuginea (TA) was measured using ImageJ by drawing a straight line from the ovarian cortex to the end of the fibrous layer and calculated by averaging four different regions of the TA in each ovarian section. Four sections were used per ovary (*n* = 8 ovaries/experimental group, *n* = 4 sections/ovary, *n* = 4 ROI/section). The percentage of PSR-, fibronectin- and laminin-positive area was calculated using four different sections per ovary and performed using the threshold tool in ImageJ (*n* = 8 ovaries/experimental group, *n* = 4 sections/ovary). PSR samples were further analyzed under polarized light to assess collagen fiber diameter and packing density. Collagen fiber color was quantified using a custom ImageJ macro. Briefly, the hue (color) of each pixel was determined, and a color threshold was used to isolate the three main colors seen in PSR-stained samples under polarized light: red (thick fibers), yellow (mid-sized fibers) and green (thin fibers). The thresholds on the hue histogram were set as previously described (Ouni *et al.* 2020, Grosbois *et al.* 2023): red 2–9, yellow/orange 10–38, and green 24–135. The relative percentage of each color was calculated by dividing the pixel count of each color by the total pixel count for each image.

### Preantral follicle isolation and culture

Ovarian cortical slices were cut from the ovarian surface using a surgical blade, and visible preantral follicles between 200 and 300 μm in diameter were mechanically dissected with 25-gauge needles. Only intact follicles with no visible signs of degeneration (darkness of the oocyte and follicular cells) and no antral cavity were selected for culture. Follicles were placed individually into 96-well V-bottomed culture plates (Corning Costar Europe, The Netherlands) in 150 μL of culture medium (McCoy’s 5a medium with bicarbonate supplemented with HEPES (20 mM; Invitrogen Ltd, UK), glutamine (3 mM; Invitrogen Ltd, UK), BSA (1 mg/mL; A9418 Sigma-Aldrich), penicillin G (0.1 mg/mL), streptomycin (0.1 mg/mL), transferrin (2.5 μg/mL), selenium (4 ng/mL), human insulin (10 ng/mL), ascorbic acid (50 μg/mL), human recombinant Activin A (100 ng/mL) and human recombinant FSH (10 ng/mL) (all obtained from Sigma-Aldrich, UK, unless otherwise stated)). Follicles were cultured individually for 15 days at 37°C in humidified air with 5% CO_2_. Half of the culture medium was replaced at 2-day intervals, and follicle diameter measurements and integrity were tracked at day 0, day 8 and day 15. Follicle diameter was obtained by averaging the two perpendicular measures from the basement membrane. Extruded cumulus-oocyte complexes (COCs) were characterized by the rupture of the basement membrane during culture and the release of the COC, while atretic follicles were recognized by a darkening of the oocytes and/or surrounding granulosa cells and a growth arrest.

### Statistical analysis

Statistical analysis was performed using SPSS v25 (IBM Corp., USA). Homogeneity of variance was verified by the Levene test and Student’s *t*-test or Mann–Whitney test were used to determine significance between the two experimental groups. Kruskal–Wallis one-way ANOVA and Dunn’s post hoc tests and univariate general linear models were used for the assessment of the follicular developmental potential *in vitro*. Data are presented as scatter plots or mean ± SD. A value of *P* < 0.05 was considered statistically significant.

## Results

### Seasonal effects on ovarian morphometry and follicular development *in vivo*

**Figure 1:**
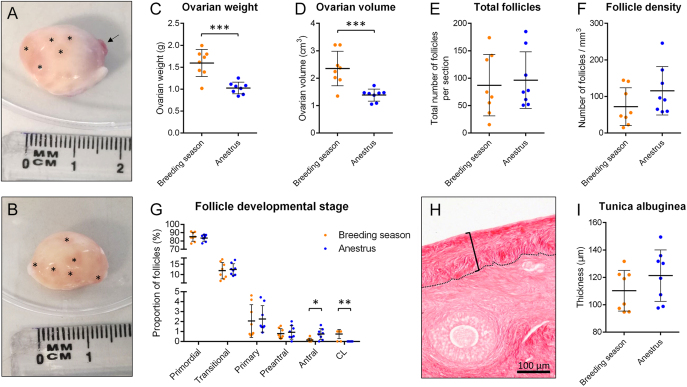
Ovarian morphometry and follicle dynamics. (A and B) Representative images of an ewe’s ovary during the breeding season (A) and during anestrus (B). Asterisks indicate visible antral follicles, and the black arrow indicates a corpus luteum. (C, D, E, F, G) Quantification of ovarian weight (C), ovarian volume (D), total follicle number per section (E), follicular density (F) and follicle developmental stage (G). *n* = 8 ovaries per experimental group. (H) Delimitation of the tunica albuginea (TA) within a PSR-stained sheep ovary section. Scale bar = 100 μm. (I) Graph showing the thickness of the TA per experimental group. *n* = 8 ovaries/group, *n* = 4 sections/ovary, and *n* = 4 ROI/section. *P < 0.05, **P < 0.01, ***P < 0.001.

At the macroscopic level, large antral follicles were visible in ovaries from both reproductive phases, but six out of the eight ovaries (75%) collected during the breeding season contained a corpus luteum, compared to none in those retrieved during anestrus (*P* = 0.010; Fig. 1A and B). Ovarian weight and volume during anestrus were reduced by 36 and 41%, respectively, compared to the breeding season (*P* < 0.001; Fig. 1C and D). The number of follicles per section and follicular density were similar for both seasons (*P* = 0.735 and *P* = 0.166, respectively; Fig. 1E and F). No difference was observed in the proportion of preantral follicles and earlier stages of development, but the percentage of antral follicles was significantly increased during anestrus compared to the breeding season (*P* = 0.028; Fig. 1G). The thickness of the TA, the connective tissue layer located beneath the surface epithelium, remained comparable in both groups (*P* = 0.215; Fig. 1H and I).

### Localization of ECM-related proteins in ovine ovaries

**Figure 2:**
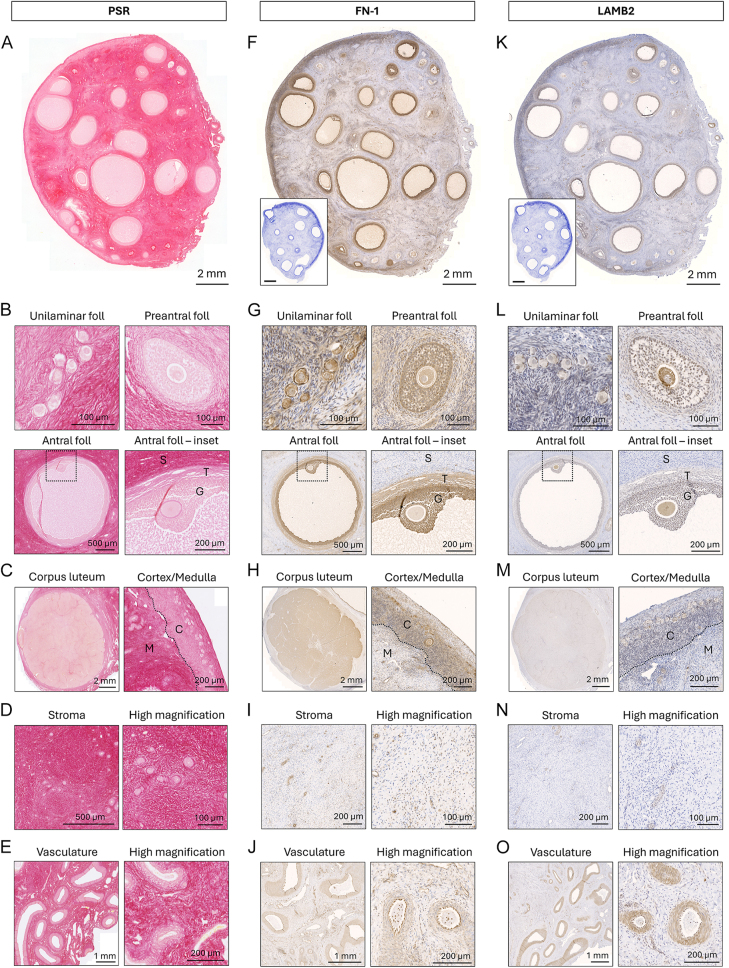
Expression and localization of collagen, fibronectin and laminin proteins in sheep ovaries. (A, F, K) Representative images of a sheep ovarian section following collagen staining with PSR (A) or following immunostaining against FN-1 – fibronectin (F) and LAMB2 – laminin (K). Collagen fibers stain in red; fibronectin and laminin in brown. Scale bar = 2 mm. Insets in F and K show negative controls of immunohistostaining; scale bar = 2 mm. (B, C, D, E) PSR staining in ovarian follicles (B), in the corpus luteum and at the cortex-medulla junction (C), in the stroma (D) and in the vasculature (E). (G, H, I, J) FN-1-staining in ovarian follicles (G), in the corpus luteum and at the cortex-medulla junction (H), in the stroma (I) and in the vasculature (J). (L, M, N ,O) LAMB2 staining in ovarian follicles (L), in the corpus luteum and at the cortex-medulla junction (M), in the stroma (N) and in the vasculature (O). G, granulosa (cumulus) cell layer; T, theca cell layer; S, stroma; C, cortex; M, medulla.

Localization and distribution patterns of collagen, fibronectin and laminin were investigated at the protein level by performing PSR staining and immunohistochemistry targeting FN-1 and LAMB2 antigens, respectively (Fig. 2). Collagen was localized to the ovarian stroma and in the periphery of follicles, and was present in abundance within the TA (Figs 1H and 2A). Collagen fibers surrounded unilaminar follicles and formed a thick layer around growing follicles (Fig. 2B). Collagen deposition was uneven in the stroma, characterized by a spatial distribution in the cortex, being highly expressed in the TA but decreased towards the medulla, with irregular patches of positive staining in the medulla (Fig. 2C and D). In contrast, collagen staining was scarce in corpora lutea and present at low levels in the vasculature (Fig. 2C and E). Fibronectin immunolocalized to the follicular compartment at all developmental stages, forming a dark region outlining the basement membrane and defining the barrier between the follicle and the stroma, and to a lesser extent to the stroma (Fig. 2F). FN-1 staining occurred most intensely in the granulosa cell compartment, from the primordial stage onwards, while the staining pattern appeared weaker in theca cells, initially arising in preantral follicles and increasing in intensity as the follicle developed (Fig. 2G). Corpora lutea were also positively stained for FN-1 (Fig. 2H). Fibronectin was present throughout the ovine ovarian stroma, but was particularly located in the cortex compared to the medulla, and in close association with the vasculature (Fig. 2H, I, J). Laminin was detected in the follicles and stroma of the sheep ovary, although the intensity of staining was relatively weak compared to the other ECM components assessed (Fig. 2K). Laminin was immunoreactive in the ooplasm from preantral follicles onwards, and in the granulosa cells of primary, secondary and antral follicles with increased staining in antral follicles (Fig. 2L). Laminin was positive at low levels throughout the theca cells with linear staining encircling each follicle, dividing the stromal compartment from the follicle unit in larger follicles. LAMB2 staining was minimal in the stroma, although staining appeared slightly stronger in the cortical region of the ovary compared to the medulla, and positive signals were detected in the vasculature (Fig. 2M, N, O).

### The ovarian ECM is dynamically remodeled across the reproductive season

**Figure 3:**
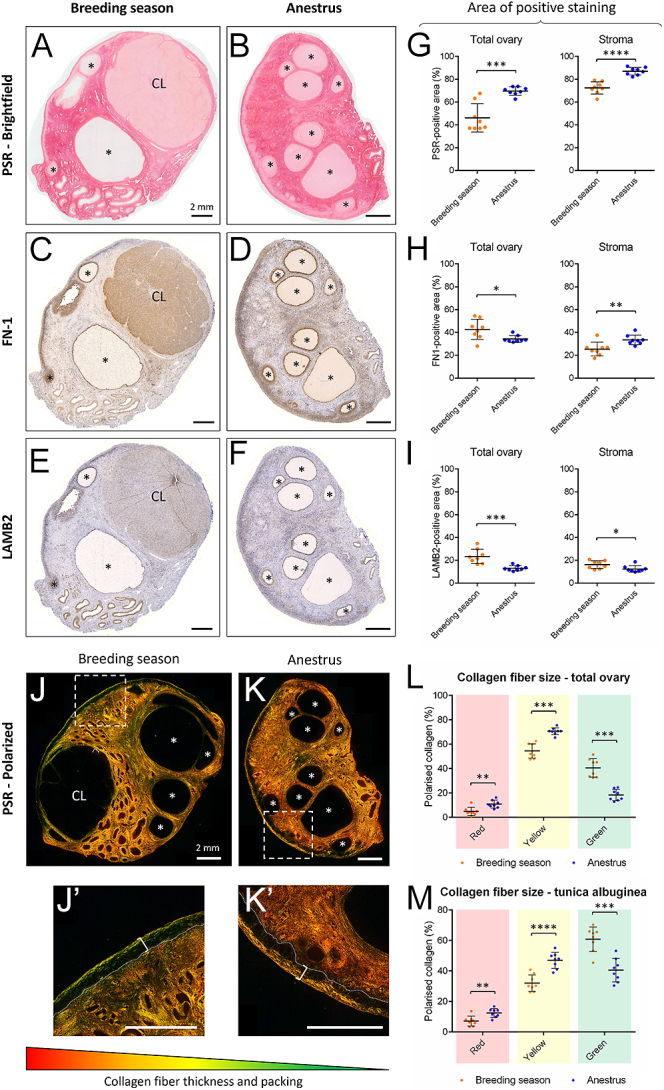
Expression of ECM-related proteins within the sheep ovary according to the reproductive season. (A, B, C, D, E, F) Representative sheep ovary sections stained with PSR (A and B) or targeted with antibodies against fibronectin (C and D) or laminin (E and F) during the breeding season (A, C, E) and anestrus (B, D, F). Asterisks indicate antral follicles; CL indicates corpora lutea. Scale bar = 2 mm. (G, H, I) Quantification of the percentage of positive area in the total ovary (left panel) and in the stroma (right panel) following PSR (G), FN-1 (H) and LAMB2 (I) staining. *n* = 8 ovaries/group, *n* = 4 sections/ovary. (J and K) Representative PSR-stained sheep ovary sections during the breeding season (J) and anestrus (K), visualized under polarized light. J′ and K′ indicate the insets of J and K, respectively, focusing on the tunica albuginea (TA; bracket). Scale bar = 2 mm. (L and M) Quantification of the relative percentage of each collagen color type in the total ovary (L) and in the TA (M). *n* = 8 ovaries/group, *n* = 4 sections/ovary. *P < 0.05, **P < 0.01, ***P < 0.001, ****P < 0.0001.

ECM remodeling across the reproductive season was evaluated by tracking the dynamics of collagen, fibronectin and laminin, both during the breeding season and at anestrus in entire ovarian cortical sections (Fig. 3A, B, C, D, E, F). At the whole ovary level, collagen deposition was higher in ovaries collected during anestrus than those collected during the breeding season, significantly increasing from 46.2% ± 12.4% positive staining during the breeding season to 69.6% ± 3.7% at anestrus (*P* = 0.001) (Fig. 3G). In contrast, both fibronectin and laminin expression were decreased during anestrus, dropping from 42.7% ± 8.8% positive staining during the reproductive season to 34.3% ± 3.0% at anestrus (*P* = 0.028), and from 23.4% ± 6.3 to 13.0% ± 2.2% (*P* = 0.007), respectively (Fig. 3H and I). Given that both the follicular compartment and corpora lutea were, to a variable extent, positively stained for all three markers and that the number of antral follicles and the presence of corpora lutea were dependent on the reproductive phase, we also quantified collagen, fibronectin and laminin content at the stroma level only. Both stromal collagen and fibronectin peaked during anestrus compared to the breeding season, rising from 72.4% ± 5.4% positive area during the breeding season to 87.0% ± 3.3% during anestrus (*P* < 0.0001), and from 25.4% ± 6.0 to 33.5% ± 4.3% positive area (*P* = 0.007), respectively, while stromal laminin slightly reduced from 16.1% ± 3.4 to 12.4% ± 2.8% positive area (*P* = 0.032). Collagen fibers’ thickness and packing density were further assessed under polarized light to evaluate collagen micro-scale structure (Fig. 3J and K). The thickest (red) fibers were the least abundant in ewes’ ovaries and represented ∼8% of total collagen, versus ∼63% and ∼29% for the mid-sized (yellow) and thin (green) fibers, respectively (Fig. 3L). All fiber types were dynamically remodeled through the reproductive season, with a significant increase of both thick and mid-sized fibers during anestrus compared to the breeding season (thick fibers: *P* = 0.003; mid-sized fibers: *P* < 0.001), while the proportion of thin fibers dropped (*P* < 0.001). Similar features were observed in the collagen fibers from the TA, characterized with a higher proportion of thick and mid-sized fibers during anestrus (*P* = 0.004 and *P* < 0.0001, respectively; Fig. 3J′, K′, M). Taken together, these results suggest the presence of a stiffer microenvironment during anestrus.

### Seasonal pattern of follicular integrity, survival and growth *in vitro*

**Figure 4:**
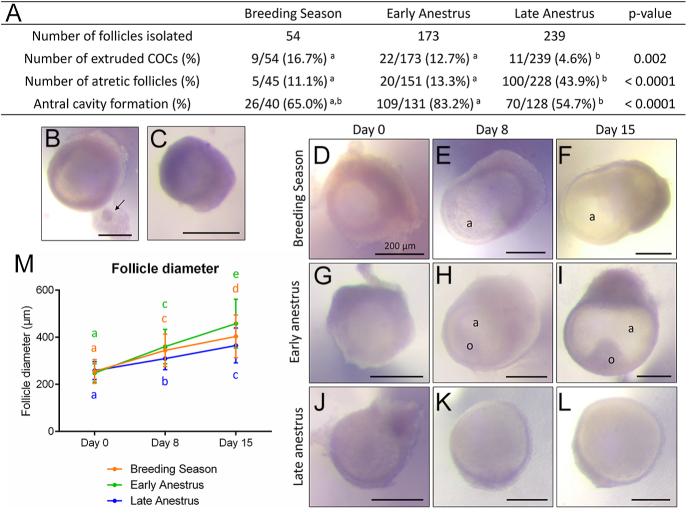
Growth patterns of isolated sheep preantral follicles during 15 days of culture. (A) Characteristics of the isolated preantral follicles following 15 days of culture. (B and C) Representative images of a follicle with an extruded cumulus–oocyte complex (COC); the arrow shows the oocyte (B) and an atretic follicle (C). Scale bar = 200 μm. (D, E, F, G, H, I, J, K, L) Representative images of preantral follicles isolated during the breeding season (D, E, F), during early anestrus (G, H, I) or during late anestrus (J, K, L) following 0 (D, G, J), 8 (E, H, K) and 15 (F, I, L) days of culture. a, antrum; o, oocyte. Scale bar = 200 μm. (M) Follow-up of the diameter of the healthy follicles (non-extruded, non-atretic) at 0, 8 and 15 days of culture. Different letters indicate statistical significance. *n* = 40 follicles in the breeding season group, 131 in the early anestrus group and 128 follicles in the late anestrus group.

The developmental potential of preantral follicles isolated during the breeding season or at anestrus was also assessed during a 15-day culture. Initial analysis indicated that follicles from the anestrus group behaved very differently according to the date of retrieval despite the exact same protocol of culture applied for most of the parameters assessed. We thus decided to conduct a subgroup analysis, based on this evidence of seasonal heterogeneity and on biological plausibility, as ovaries obtained during early anestrus might still be under the influence of steroid hormones and could, to some extent, have in-between intrinsic capacities compared to follicles retrieved during the breeding season and during late anestrus. Hence, anestrus was divided into early anestrus group (retrieved from mid-April till mid-May) and late anestrus group (retrieved from mid-May till the end of June). A total of 54, 173 and 239 follicles in the breeding season, the early anestrus and the late anestrus group, respectively, were isolated and cultured individually. 5–17% of follicles lost their structural integrity by the end of the culture, with follicles from the late anestrus group significantly less likely to dismantle (Fig. 4A). Follicle loss occurred mainly through the breakdown of the basal membrane and opening of the follicle releasing the COC (Fig. 4B). Follicle survival was significantly decreased in the late anestrus group (56%) compared to those from the breeding season and early anestrus groups, which displayed similarly high rates of survival (87–89%) following the 15-day culture period (Fig. 4A and C). Morphologically, follicles from all groups significantly grew over time and were able to form an antral cavity, although those isolated during late anestrus had a reduced rate of antrum formation compared to those retrieved during early anestrus (Fig. 4A, D, E, F, G, H, I, J, K, L). The mean diameter of the healthy follicles (non-extruded, non-atretic; *n* = 299) at the onset of culture was 253.6 μm ± 40.6 μm. Between day 0 and day 15 of culture, follicles retrieved during the breeding season, early anestrus or late anestrus showed a 1.6-, 1.8- and 1.4-fold increase in diameter, respectively, and reached a final diameter of 403.6 μm ± 90.8 μm, 458.2 μm ± 103.6 and 365.0 μm ± 74.3 μm, respectively (*P* < 0.0001; Fig. 4M). Follicle diameter was found to correlate with both the season and the culture period (*P* < 0.0001), with an accelerated growth during early anestrus compared to the breeding season, while that of late anestrus was slowed down.

## Discussion

There has been a growing interest in investigating the ovarian stroma, particularly the physical, biochemical and mechanical cues that exist between ovarian cells and their microenvironment, which regulate their multi-directional interactions (Thorne *et al.* 2015, Kinnear *et al.* 2020, Matsuzaki 2021, Biswas *et al.* 2022, Fiorentino *et al.* 2023). However, limited information is available regarding how the ECM regulates follicle dynamics and tissue patterning during ovarian function. The sheep is a seasonal breeder, a phenomenon that involves changes in ovarian biology, including reversible fertility. Using this animal model, we observed differences in ovarian morphometry according to the season, which coincided with a dynamic remodeling of the ovarian ECM. The breeding season was characterized by complete cycles of folliculogenesis and ovulation, while anestrus was marked by an arrest of follicle development at the antral stage and anovulation. At the stroma level, ewe ovaries collected during anestrus displayed increased collagen deposition and altered ECM composition, which creates a biomechanically non-permissive environment that may contribute to inhibiting late follicle growth and ovulation and altering ovarian shape. In addition, preantral follicles isolated from their native environment displayed a season-dependent pattern of follicular integrity, survival, antrum formation and growth. Altogether, these data provide evidence that seasonal breeding impacts and regulates ovarian follicles and their surrounding microenvironment.

Reduction in ovarian mass, lower sex steroid hormone production and anovulation characterize females outside of the breeding season (Beltran-Frutos *et al.* 2022). In this study, the ovaries of anestrus ewes showed signs of atrophy, with decreased weight and size. Similar features have been reported in rodents in response to photoperiod (Moffatt-Blue *et al.* 2006, Pal *et al.* 2022). Despite this phenomenon, anestrus ovaries remained active, with follicle numbers and proportion of follicles up to the preantral stage similar to the breeding season. The rise in the proportion of antral follicles may reflect a change in follicular responsiveness to gonadotropins and/or ovarian steroids, particularly estrogens, despite their low levels. In contrast, no corpora lutea were visible during anestrus, indicating that the lack of gonadotropin support prevents the final stage of folliculogenesis and ovulation. These data match with previously published works in ewes (Brand and de Jong 1973, Bartlewski *et al.* 1998, Di *et al.* 2014).

Although the ovarian ECM has been characterized for several domesticated livestock animals, including porcine (Henning *et al.* 2019, Parkes *et al.* 2021, Pennarossa *et al.* 2022) and bovine species (Zhao & Luck 1995, McArthur *et al.* 2000, Parkes *et al.* 2021), as well as humans (Ouni *et al.* 2019, Ouni *et al.* 2020, Grosbois *et al.* 2023), little data are available for the sheep. Previous work from Huet and colleagues showed that ECM components are present in the basement membrane as well as around granulosa cells, thecal cells and in the follicular fluid of ovine follicles, and that follicular growth and atresia in the sheep are accompanied by changes in ECM content and localization (Huet *et al.* 1997). This study further establishes that the ECM content of the ewe ovary changes during follicular development in a spatiotemporal fashion. Spatially, we observed compartment-specific collagen, fibronectin and laminin localization, consistent with previous data reported in mammals (Berkholtz *et al.* 2006, Hummitzsch *et al.* 2019, Parkes *et al.* 2021, Grosbois *et al.* 2023). Interstitial collagen provides stromal cells with structural support and follicles with a gradually softening environment from the cortex to the medulla that may regulate their activation and growth (Woodruff & Shea 2007), whereas perifollicular fibronectin and laminin, which both serve as connectors within the ECM (Dalton & Lemmon 2021), may contribute to reinforce and stabilize the local ECM and support follicle development through the binding of growth factors. Temporally, the ECM was dynamically remodeled across season, with the establishment of a stiffer environment at anestrus characterized by increased stromal collagen and fibronectin expression and thicker ovarian collagen fibers, while stromal laminin content decreased. Anestrus was also correlated with thicker and more packed collagen fibers in the TA, which could hinder the action of proteolytic enzymes responsible for the rupture of the ovarian surface and thus impede ovulation. These data match results obtained in hamster and sheep ovaries, in which season also impacted the expression of ECM and matrix-associated genes (Chen *et al.* 2012, Leon *et al.* 2020, Wei *et al.* 2022). Future experiments should investigate the mechanical properties of the sheep ovary during the breeding season and anestrus in order to clarify the consequences of such structural changes at a functional level.

How ECM remodeling in relation to the stage of the breeding season is regulated is unclear. The switch from breeding to anestrus season in the ewe is associated with a marked change in the GnRH neurosecretory system – the LH pulse-generating system, although active, is compromised because both frequencies of GnRH and LH are extremely low (Karsch *et al.* 1993, Chemineau *et al.* 2008). In addition, the levels of LH, FSH, estradiol (E2) and progesterone (P4) drop (Di *et al.* 2014, Zhai *et al.* 2018). Estrogen and FSH receptors (ERα, ERβ and FSHR, respectively) have been shown to be upregulated in sheep ovaries during anestrus, enhancing ovarian responsiveness to E2 and FSH and allowing follicular waves to occur while exerting a strong negative feedback on the hypothalamic–pituitary–ovarian axis to maintain anestrus (Li *et al.* 2019). Of interest, ERβ has been demonstrated to regulate ECM composition in the mouse ovary, including collagen and laminin content (Inzunza *et al.* 2007, Zalewski *et al.* 2012). ECM turnover is also regulated by the action of matrix metalloproteinases (MMPs) and their inhibitors, tissue inhibitors of metalloproteinases (TIMPs), themselves modulated by hormonal signals (Curry *et al.* 2003). It is thus plausible that the seasonal fluctuations of sex hormones may modulate the extracellular environment which, together, regulate follicle dynamics. Besides, the presence of corpora lutea at the ovarian surface is dependent on the reproductive season, with corpora lutea not physiologically present during seasonal anestrus. Although corpora lutea themselves do not seem directly responsible for the changes observed in follicle dynamics and ECM remodeling (Supplementary Fig. 1 (see section on Supplementary materials given at the end of the article)), it is possible that their secretion of progesterone might participate in ECM turnover by regulating the MMP/TIMP system (Curry *et al.* 2003). Importantly, this state of ovarian involution and compressive environment during anestrus is cyclic and reversible, being potentially overcome through the rise of sex hormones and the loosening of the ECM at the onset of a new breeding season. Nevertheless, it is likely that this high ECM turnover capacity declines over time, due to the age-related gradual rise in stiffness and establishment of an ovarian fibrosis (Amargant *et al.* 2020, Ouni *et al.* 2020).

*In vitro* culture of isolated preantral follicles has been successfully developed in the sheep (Cecconi *et al.* 1999, Thomas *et al.* 2003, Barboni *et al.* 2011). In this study, by removing follicles from their native microenvironment and exposing them to a standardized culture medium containing hormones and growth factors, we were able to assess whether the season-related ovarian changes had long-term effects on follicular growth. We report that preantral follicles cultured for 15 days significantly grew and reached the antral stage, as reported in other studies (Cadoret *et al.* 2017, Monte *et al.* 2019, Silva *et al.* 2021). However, preantral follicles showed a seasonal pattern of follicular integrity, survival, antrum formation and growth, with a decreased rate of extruded COC, a higher proportion of atresia and a smaller follicular diameter in the late anestrus group compared to breeding season and early anestrus, while follicles retrieved during early anestrus exhibited a quicker development. We speculate that during early anestrus, follicles retain their ability to grow despite the drop of sex hormones and that their removal from a rapidly stiffening environment may rescue their developmental potential. In contrast, follicles retrieved during late anestrus may have decreased intrinsic growth capacities, creating less pressure on the follicular wall and thus being less likely to break. Previous research has also shown a season-dependent *in vitro* developmental competence of sheep oocytes, with higher developmental competence observed during the breeding season (Mara *et al.* 2014, Ebrahimi *et al.* 2024).

One limitation of the study is that we could not control for age and breed from which the ovaries were harvested, although we know that the company primarily processes Texel and Scottish Blackface sheep breeds as well as Texel/Scottish Blackface crossbreds, and that the animals had reached puberty. Moreover, reasons for culling were unknown, whether for reproductive-related reasons (parity, lactation stage, reproductive performance, lambing season and milk yield) or advanced age, which could influence the results, or for diseases, poor body condition, lameness and production losses. Despite that, the biological replicates were often close to one another and did not overlap with those from the other experimental group, and statistical difference was achieved for some of the parameters assessed.

Altogether, these results offer a first look at the dynamic ECM remodeling that occurs across reproductive seasonality in sheep, modulating the microenvironment to potentially regulate follicle growth and ovulation. Understanding the molecular mechanisms underlying seasonal breeding has significant implications for improving oocyte yield and embryo production for agricultural purposes. It may also reveal key aspects for restoring ovarian function in women, particularly in the case of ECM-related ovarian disorders, which have been described in patients with polycystic ovarian syndrome, and ovarian changes induced by testosterone treatment for transgender patients or by aging (Shah *et al.* 2018, Amargant *et al.* 2020, Bailie *et al.* 2023). Besides, improvement of current *in vitro* growth systems or the development of the artificial ovary technology calls for a clear understanding of the native environment and how scaffolding components can influence folliculogenesis.

## Supplementary materials



## Declaration of interest

The authors declare that there is no conflict of interest that could be perceived as prejudicing the impartiality of the research reported.

## Funding

This study was supported by the Medical Research Councilhttps://doi.org/10.13039/501100000265 (grant MR/T025654/1).

## Author contribution statement

JG, VS and EET conceived the study. JG and VS performed the experiments, collected and analyzed the data. AMG contributed to the data collection. JG and EET interpreted the data. HMP and EET acquired funding support. JG wrote the manuscript. All authors edited and approved the final version of the manuscript.
