# Supplementary material for: Reproductive seasonality influences follicle dynamics and the ovarian extracellular matrix structural properties in ewes
Source: Reproduction. 2025 May 22;169(6):e250010. doi: 10.1530/REP-25-0010 (PMC12100507; doi:10.1530/REP-25-0010)
Supplement: Supplementary file 1 [file supplementary_materials.pdf]

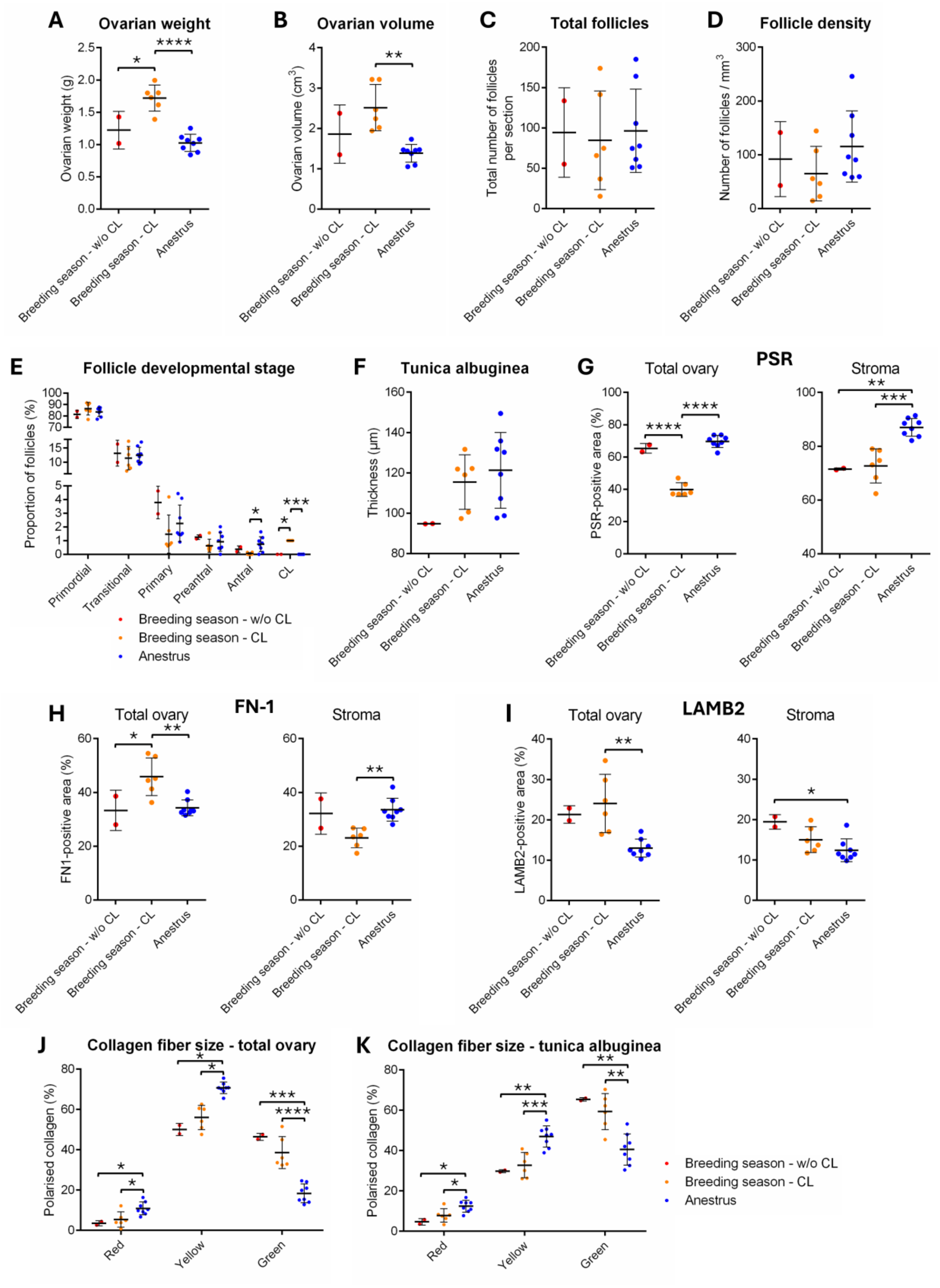

Supplemental Figure 1: The presence of corpora lutea (CLs) during the breeding season is not the main driver of the changes observed in follicle dynamics and ECM turnover. Ovaries retrieved during the breeding season were further divided according to the presence or not of CL at the ovarian surface (labelled as CL and w/o CL, respectively) and data related to

ovarian morphometry, follicle dynamics and ECM turnover were re-analysed. A-F: quantification of ovarian weight (A), ovarian volume (B), total follicle number per section (C), follicular density (D), follicle developmental stage (E) and the thickness of the tunica albuginea (F). G-I: quantification of the percentage of positive area in the total ovary (left panel) and in the stroma (right panel) following PSR (G), FN-1 (H) and LAMB2 (I) staining. J,K: quantification of the relative percentage of each collagen colour type in the total ovary (J) and in the tunica albuginea (K). Breeding season without CL, N = 2; Breeding season with CL, N = 6; Anestrus, N = 8. *P < 0.05, **P < 0.01, ***P < 0.001, ****P < 0.0001.
